# Musculoskeletal injury research in sub-Saharan Africa: a ten-year bibliometric analysis of research outputs from Malawi, South Africa, and Tanzania

**DOI:** 10.1302/2633-1462.73.BJO-2025-0289.R1

**Published:** 2026-03-27

**Authors:** Robyn Waters, Shahd Osman, Maritz Laubscher, Sithombo Maqungo, Nyengo Mkandawire, Billy Haonga, George Njambilo, Matthew L. Costa, Linda Chokotho, Simon Matthew Graham, National Institute for Health and Care Research Global Health Research Group on Global Injury

**Affiliations:** 1 Orthopaedic Research Unit, Division of Orthopaedic Surgery, Department of Surgery, Groote Schuur Hospital, University of Cape Town, Cape Town, South Africa; 2 Division of Orthopaedic Surgery, Department of Surgery, Groote Schuur Hospital, University of Cape Town, Cape Town, South Africa; 3 Federal Ministry of Health, Khartoum, Sudan; 4 Division of Global Surgery, University of Cape Town, Cape Town, South Africa; 5 Kamuzu University of Health Sciences, Blantyre, Malawi; 6 Muhimbili University of Health and Allied Sciences, Dar es Salaam, Tanzania; 7 Oxford Trauma and Emergency Care, Nuffield Department of Orthopaedics, Rheumatology & Musculoskeletal Sciences, University of Oxford, Oxford, UK; 8 Center for Clinical and Biological Sciences Research (CCBSR), Academy of Medical Sciences, Malawi University of Science and Technology, Thyolo, Malawi; 9 Orthopaedic Research Unit, Division of Orthopaedic Surgery, Department of Surgery,, Groote Schuur Hospital, University of Cape Town, Cape Town, South Africa, Cape Town, South Africa

**Keywords:** Bibliometric analysis, Low-income countries, Musculoskeletal, Orthopaedic, Research, Trauma, Fracture, Sub-Saharan Africa, Injury, musculoskeletal injuries, bibliometric analysis, lower limb injuries, Traumatic injury, strength, randomized controlled trial, clinical trials, Bone fracture, traumatic brain injury, Traumatic fractures

## Abstract

**Aims:**

Musculoskeletal (MSK) injuries pose a significant health burden across sub-Saharan Africa (SSA). Despite this, regional research output is limited, constrained by insufficient infrastructure, limited funding, and underdeveloped research capacity. This bibliometric analysis examined research outputs and collaboration patterns in MSK injury research across three SSA countries, representing a spectrum of income levels: low, lower-middle, and upper-middle income.

**Methods:**

MSK injury research articles from Malawi (MLW), South Africa (SA), and Tanzania (TZN), published between January 2014 and April 2024, were identified using Web of Science and Scopus databases, and cross-referenced with ResearchGate. Data were analyzed descriptively in Excel, and institutional coauthorship and collaboration networks were mapped using VOSviewer.

**Results:**

A total of 329 articles were published across MLW (n = 98), SA (n = 141), and TZN (n = 90) between January 2014 and April 2024. We report a steady increase in the number of publications from 2014, with a research focus on fracture management, outcomes, and lower limb injuries. Most of the research was published in partnership with high-income countries (HICs) (SA: 51%, MLW: 98%, TZN: 87%), with high-income country (HIC) institutions dominating first and last authorship in MLW (68% and 61%) and TZN (59% and 69%) publications. Most studies were descriptive and based on secondary records. Institutional networks showed strong regional collaboration in SA, international focus in MLW, and limited connectivity in TZN.

**Conclusion:**

MSK injury research is increasing across MLW, SA, and TZN, but remains largely HIC-led and descriptive, particularly in lower-income countries. Strengthening local leadership, regional collaboration, and research capacity is essential for more sustainable and context-specific evidence generation.

Cite this article: *Bone Jt Open* 2026;7(3):455–464.

## Introduction

Sub-Saharan African (SSA) countries have higher incidences of musculoskeletal (MSK) injuries compared with any other region in the world, and populations are expected to double by 2050. This is expected to significantly increase the burden of MSK injuries, overwhelming already limited trauma care and rehabilitation services.^1-3^ The amount of funding, infrastructure, and research dedicated to MSK injury care is disproportionally low in comparison to other significant global health problems, such as communicable diseases, especially in low-middle income countries (LMICs).^4^ This reflects both a disease-specific imbalance and a broader global disparity in research investment allocation, as captured by the 10/90 gap, where only 10% of global research funding addresses the needs of the populations bearing 90% of the health burden. The underinvestment in MSK research in LMICs illustrates the need for more equitable funding across both health conditions and regions.^1,5^

There are numerous barriers and challenges to undertaking MSK injury research across SSA and other LMICs, including limited research funding and support, resources, infrastructure, training, and capacity.^6-10^ As a result, little is known about the true prevalence and burden of MSK injuries, prevention strategies, healthcare provision and resource use, longer-term consequences, and the wider impact of MSK injuries in LMICs.

High-level evidence studies, such as longitudinal observational or clinical trials, are critical to provide robust data and support evidence-based decision making. However, they are very costly, and necessitate skilled human resources and substantial financial investment. These barriers, along with issues such as data accessibility, regulatory requirements, and infrastructure limitations, significantly impact the overall production of research, especially in LMICs.^11-13^ In addition, there is under-representation of African researchers in global health publications due to a complex mix of structural, systemic, and institutional factors.^14^ This is despite international collaborations, which can bridge the gap by enhancing infrastructure and capacity-building for local researchers. Therefore, it is now common practice to engage in institutional collaborations to produce high-impact research.^11,15-17^

Research activity and output can be assessed by considering the volume of research articles produced by or associated with a country or region.^18^ It is estimated that only 1.8% of the publications in the Web of Science (WOS) bibliographic database originate from Africa, with Egypt and South Africa (SA) accounting for the majority of these contributions.^11^ This is despite Africa being home to approximately 18% of the global population, highlighting a substantial imbalance in global research output. A baseline assessment to determine the status of MSK research in SSA remains to be addressed. Thus, the aim of this study was to analyze the MSK injury research outputs across Malawi (MLW), South Africa (SA), and Tanzania (TZN) in the past ten years. These three countries span low to upper-middle and low-middle income levels, respectively, offering a representative snapshot of SSA’s socioeconomic diversity. Through this research, our objective is to share current MSK research focus areas and identify areas for improvement. In addition, we hope to find ways to drive collaborative initiatives that will enhance the quality and output of MSK injury research in SSA, leading to more evidence-based care and improved patient outcomes.

## Methods

### Study design

We conducted a bibliometric analysis of MSK injury research articles published from MLW, SA, and TZN in the decade from 2014 to 2024. These three countries represent a spectrum of income classifications: low, lower-middle, and upper-middle income, respectively, and therefore provide a more representative reflection of the socioeconomic diversity within SSA. Additionally, MLW, SA, and TZN were chosen based on existing research collaborations within our Global Injury research Group (GIG), established through a National Institute for Health and Care Research (NIHR)-funded programme (NIHR155559). Ethical approval for our research was obtained from the University of Cape Town Human Research Ethics Committee (HREC Ref: 212/2024).

### Inclusion and exclusion criteria

Articles were included if they concerned MSK trauma, injury and/or fracture, experienced by both adults and children. The research material origin was not part of the inclusion criteria. Articles were excluded if they concerned any injury other than MSK trauma and fracture (e.g. burns, traumatic brain injury) or injury experienced by animals. Letters or correspondence were also excluded.

### Search strategy

The Web of Science (WOS) database (Clarivate) and Scopus (Elsevier) was searched to identify all indexed articles that met the eligibility criteria. The following keywords and Boolean terms were used: "Musculoskeletal trauma" OR "Musculoskeletal injury" OR "Musculoskeletal injuries" OR "Traumatic fractures" OR "Bone fracture" OR "Traumatic injury". The WOS was searched using ‘all databases’ and topic (TS) and Scopus included ‘all fields’. A filter was then applied per database for relevance to include country of origin (MLW, SA, and TZN), English language, custom date range (January 2014 to April 2024 for WOS and 2014 to 2024 for Scopus, respectively), articles type (article, review, observational study, case report, and clinical trial) and corresponding data were exported into Excel (Excel for Microsoft 365 MSO, v. 2501; Microsoft, USA). Results were cross-checked with major researcher profiles in each country on ResearchGate.

### Data extraction

**Fig. 1:**
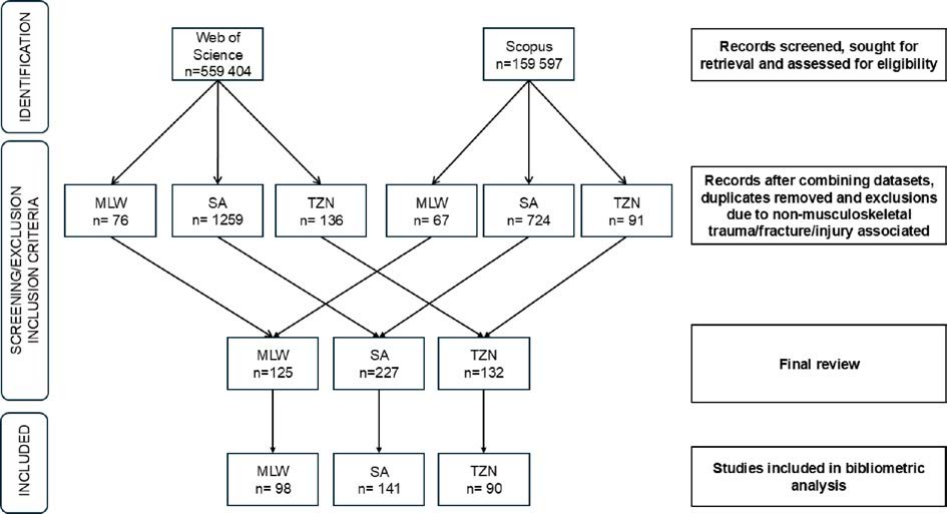
Screening process for bibliometric analysis. MLW, Malawi; SA, South Africa; TZN, Tanzania.

For every article included, the following information was extracted: journal title, abstract, geographical location and country, year of publication, citation number, and type of study. Figure 1 and the Supplementary Material give more details on the screening process. The final publications included were categorized into the following eight themes according to the focus of the article: Burden, Epidemiology, Presentation/Diagnosis, Management, Complications, Outcome, Health System, and Training and Research (see Supplementary Material Appendix 1 for definitions).

### VOSViewer

VOSViewer (v. 1.6.20; Leiden University, Netherlands) was used to generate coauthorship network visualizations at the institutional level and to explore patterns of research collaboration. Networks were constructed using full counting, which accounts for all coauthorship links between institutions. To enhance interpretability and reduce noise from large, diffuse collaborations, we excluded publications with more than 25 contributing institutions. Furthermore, only institutions with more than one included publication were retained in the analysis, to highlight entities with sustained research activity rather than isolated contributions.

### Patient and public involvement

Patients and the public were involved in the development of the Global Injury Group’s initial funding proposal, but not directly in the design or conduct of this bibliometric study. Here, input is from local researchers, healthcare workers, and stakeholders who identified MSK injury research as a priority area. Community engagement initiative (CEI) representatives will be involved in the dissemination of results, helping to identify key messages and appropriate formats to ensure that findings are accessible and relevant to patient communities and local contexts.

### Statistical analysis

Descriptive statistics (n (%)) were used to summarize the number of research articles, citations, country collaborations, institutions, coauthorship, type and position of injury, thematic focus of research, and study designs. Subanalyses were performed to determine trends in publishing over the ten years in each of the three countries (while data through to April 2024 was included in the overall analysis, the trend analysis was limited to complete calendar years), focus areas of research, researcher affiliations (per country), output per institution in each country, and collaborative partnership efforts, both locally and globally with high-income countries (HICs). Citation rate was calculated by dividing the total number of citations by the number of publications for each country.

## Results

The initial bibliometric search identified 559,404 and 159,597 research articles from WOS and Scopus, respectively. After screening and application of inclusion and exclusion criteria, a total of 329 research articles were included for the final analysis from MLW (n = 98), SA (n = 141), and TZN (n = 90) (Figure 1).

**Fig. 2:**
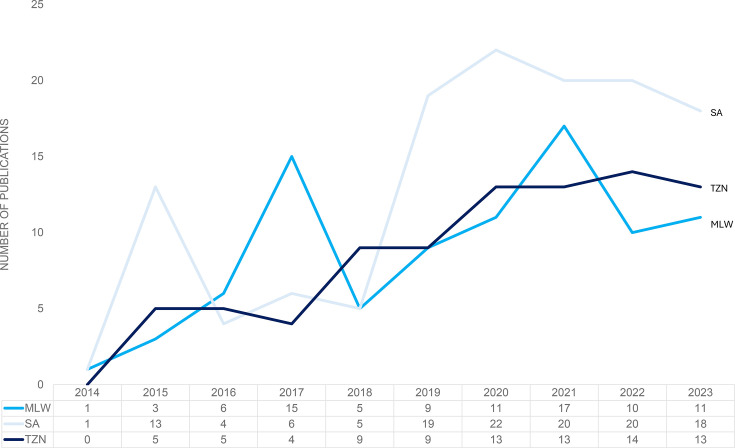
Number of publications included in the bibliometric analysis per country and ten-year publication trend for the three countries of interest. Only complete calendar years (2014 to 2023) are shown. Data for 2024 (January to April) were excluded to avoid distortion from partial-year counts. MLW, Malawi; SA, South Africa; TZN, Tanzania.

Data indicated an upward trend in the number of publications across all countries since 2014. SA experienced significant growth in 2019 onwards, MLW showed spikes in 2017 and 2021, while TZN showed more of a gradual yearly growth (Figure 2). Total citations were highest in SA (n = 1,797), followed by TZN (n = 1,199) and MLW (n = 1,113). The median citation rate for SA was 5 (IQR 2 to 14), for MLW: 8.5 (IQR 2 to 14) and for TZN: 9 (IQR 3 to 17), with 13, six, and six publications with zero citations in the three countries, respectively.

Most of the studies were descriptive in nature reporting analysis outputs from secondary data (e.g. existing medical records). Country-specific results are represented in the Supplementary Material. In terms of specific study designs, 0% (MLW), 2% (SA), and 8% (TZN) were randomized controlled trial (RCT) outputs. Longitudinal observational studies were comparatively low, with 13% (MLW), 6% (SA), and 20% (TZN) cohort studies and 0% (MLW), 1% (SA), and 1% (TZN) case-control studies. Regarding evidence syntheses, only 3% (MLW), 5% (SA), and 0% (TZN) of all included publications were systematic reviews with or without meta-analysis.

**Fig. 3:**
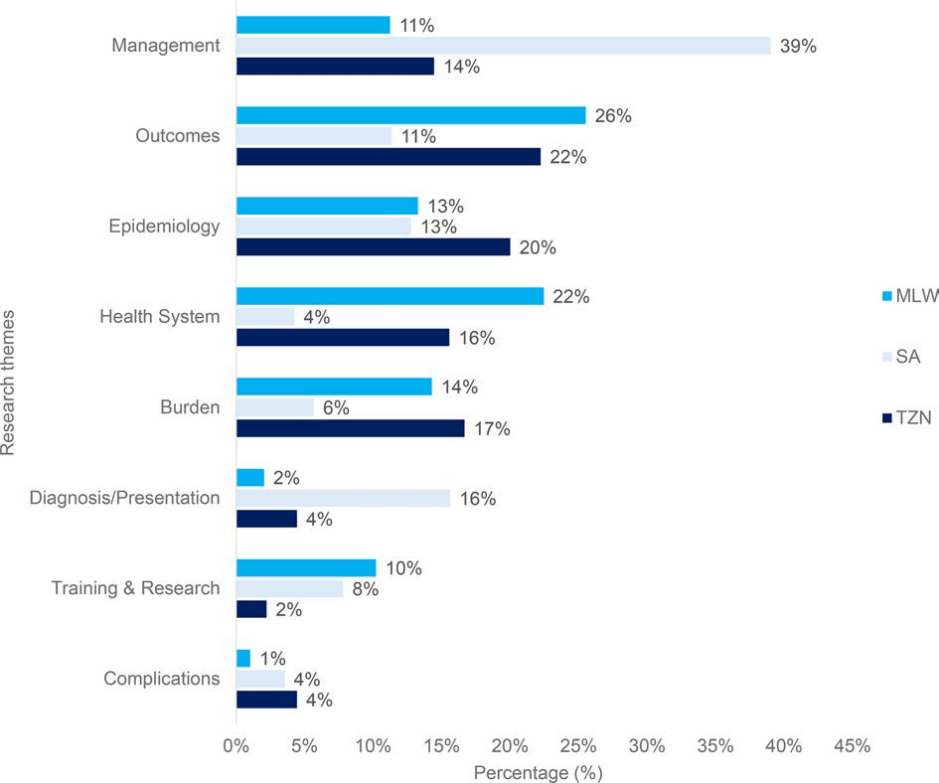
Research article themes per country of interest. ‘Burden’ refers to the number of musculoskeletal injuries which occur, as well as the overall impact that these injuries have on individuals, healthcare systems, and wider society. MLW, Malawi; SA, South Africa; TZN, Tanzania.

Overall, across the three countries, most research studies were focused on the management and outcomes of MSK injury (Figure 3), especially in lower limb injuries.

MLW had the highest rate of international collaborations, with 98% (n = 96) of its publications involving partners from other countries. This was closely followed by 87% (n = 78) and 51% (n = 72), in TZN and SA, respectively. Notably, most of these collaborations were North-South in nature. For both SA and TZN, 96% of the collaborations were with institutions from the Global North (SA: 69, TZN: 75) and only 4% with the Global South (SA: 3, TZN: 3). MLW’s collaborations were exclusively with institutions from the Global North. The UK and USA were the top two country collaborators for all three countries of interest.

We report the most frequent local and external/international institutes named as author affiliation for each of the partner countries. The top local institutes included Kamuzu Central Hospital (MLW), the University of Cape Town (SA), and Muhimbili Orthopaedic Institute (TZN), and the top external institutes included University of North Carolina at Chapel Hill (MLW), Royal Brisbane and Women’s Hospital (SA), and University of California, San Francisco (TZN).

We analyzed country affiliations of the first and last authors given the significant collaborations between Northern and Southern institutes. In SA, both first and last authors were mostly affiliated with in-country SA institutes: 64% and 60%, respectively. However, in MLW and TZN, authors were more likely to be from external institutes.

**Fig. 4:**
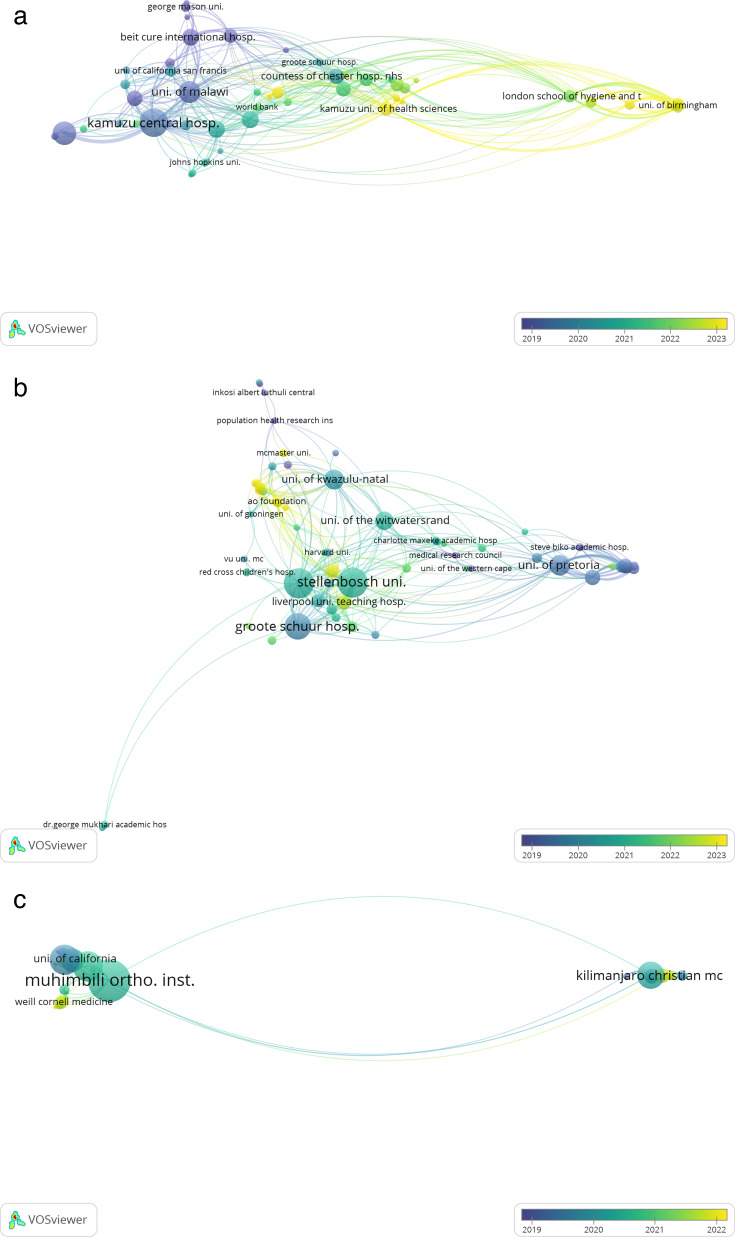
The a) Malawi, b) South Africa, and c) Tanzania institutional coauthorship network using VOSViewer (v. 1.6.20; Leiden University, Netherlands). Nodes represent individual organizations, with larger nodes indicating institutions with higher publication output or more significant collaborative ties. The positioning of nodes reflects their connections, where closely grouped nodes indicate stronger research partnerships. Links represent coauthorship connections between institutes, where each link indicates a collaborative publication. Link strength refers to the cumulative frequency of these collaborations, meaning a higher strength value signifies a stronger and more frequent partnership between entities. The number of clusters represents distinct groups of interconnected organizations within each country, reflecting the structural composition of collaboration networks.

The VOSviewer coauthorship networks revealed distinct patterns of collaboration across MLW, SA, and TZN, which are summarized in Table I and Figure 4. MLW’s network returned the highest cumulative strength (total link strength = 840), suggesting broader but more cohesive partnerships among institutions. SA’s network displayed a mix of strong and weak connections, with distinct clusters where key institutes maintain frequent collaborations (total link strength = 695). TZN’s network exhibited a more concentrated structure, where fewer organizations engage in more frequent collaboration within specific clusters (total link strength = 330).

**Table I.** Institutional collaboration and network characteristics of musculoskeletal injury research by country.

Feature*	Country		
	MLW	SA	TZN
Total institutes (n)	160	264	117
Institutes meeting threshold,† n	53	59	37
Connected clusters, n	5	7	6
Links, n	349	324	119
Total link strength	840	695	330
Network density	Moderate	High	Sparse
National hubs	Kamuzu Central Hospital, University of Malawi	Groote Schuur, Stellenbosch University, Wits University	Muhimbili Orthopaedic Institute, Kilimanjaro Christian Medical Centre
International reach	UK, USA, World Bank	UK, USA, Canada, Netherlands	USA (mainly California, Cornell)
Intra-country links	Limited	Strong	Minimal
Regional African links	Minimal	Limited	Absent
Temporal activity	Recent growth (2021 to 2023)	Consistent growth (2019 to 2023)	Early phase (2020 to 2022)

* See Supplementary Material for ‘Feature’ details.

† To enhance interpretability and reduce noise from large, diffuse collaborations, we excluded publications with more than 25 contributing institutions.

MLW, Malawi; SA, South Africa; TZN, Tanzania.

The network for MLW exhibited a moderately dense structure, with a small number of national institutions forming the core of collaborative activity. Kamuzu Central Hospital, the University of Malawi, and Kamuzu University of Health Sciences were the most prominent institutions, with frequent coauthorship links indicating sustained regional involvement in MSK research. Strong international collaborations were evident, particularly with institutions in the UK and the USA. Temporal data suggested a notable increase in collaborative activity between 2021 and 2023. However, intranational and regional (African) collaborations were limited.

The SA institutional network was the most expansive and densely connected of the three countries. Several institutions including Groote Schuur Hospital, Stellenbosch University, and the University of the Witwatersrand occupied central positions, reflecting their significant contribution to MSK research. Intra-country collaboration was strong, with frequent coauthorship observed among major academic and clinical institutions. International partnerships were also well established, spanning the UK, USA, Canada, and parts of Europe. Collaborative activity was consistently high from 2019 through 2023, indicating a mature and stable research environment. Despite its density, there was relatively little engagement with institutions in other SSA countries.

Finally, in TZN, the institutional network was comparatively sparse, with only a few institutions engaged in MSK research, with Muhimbili Orthopaedic Institute and Kilimanjaro Christian Medical Centre emerging as the primary national nodes. International collaboration was limited to a small number of USA-based partners, notably the University of California and Weill Cornell Medicine. There was minimal evidence of intra-country collaboration or regional engagement with other African institutions. Most collaborative activity appeared to have taken place between 2020 and 2022.

## Discussion

We present a summary of MSK injury-focused research from MLW, SA, and TZN over a ten-year period. Publications increased across all countries from 2014, with a focus on fracture management and outcomes (particularly of the lower limb), reflecting previously identified orthopaedic priorities.^19,20^ Less research attention was given to training, research capacity, and injury complications. Collaboration with HICs was common, especially in MLW (98%) and TZN (87%), compared with 51% in SA. Most collaborations were North-South, with the UK and USA as leading partners. In SA, first and last authors were mostly locally affiliated, while in MLW and TZN these roles were more often held by external researchers. This raises concerns about whether local researchers in these countries are being adequately supported and trained to take on leadership roles in research, particularly as first and last authorship is often indicative of research ownership and leadership capacity. Most studies were descriptive and relied on secondary data, likely due to ease of access and feasibility. Coauthorship networks were strongest in SA, with weaker linkages in MLW and TZN.

The overall citation rates in this study were relatively high, above global averages, where many publications receive few or no citations. However, the wide citation ranges reflect a highly skewed distribution, where a small number of articles, often systematic reviews or multicentre clinical trials, accounted for a large share of citations. Many other articles received few or none. As seen in other bibliometric studies, research from higher-income countries, with higher research and development investment, tends to have broader reach and higher citation impact.^21,22^ These findings highlight both the growing visibility of MSK research in SSA and the continued influence of study type, authorship profile, and level of international collaboration on citation performance.

Research patterns suggest that income level influences research leadership and collaboration. SA, an upper-middle-income country, showed stronger local authorship and institutional networks, while MLW and TZN, both lower-income, relied more on international partnerships. This supports the idea that as countries develop economically, reliance on external partners decreases, and authorship becomes more locally led. Targeted investment in lower-income settings may help build independent, sustainable research capacity. MSK injury research in SSA is increasing, often with HIC partners, reflecting growing capacity. Key enablers include local resources and collaboration,^23^ while funding, time, and training barriers remain.^5,24^ Despite challenges, opportunities for impact, mentorship, and funding continue to drive LMIC researchers.^5,10^ To build sustainable MSK research capacity in SSA, it is crucial to strengthen domestic investment, foster equitable partnerships, and expand access to training and dissemination. Leveraging regional and international networks can support high-quality research that informs clinical practice and health policy.^25,26^

Few articles reported RCTs or longitudinal studies, partly due to high patient loss to follow-up in LMICs driven by unemployment, travel distance, and cost.^27^ Training, research capacity, and injury outcomes or complications were less common research areas, yet training underpins research productivity, and complications and infections are clinically urgent. Outcome studies require follow-up and funding, both of which are noted challenges in the region.

Previous bibliometric analyses of surgical research across LMICs have demonstrated a steady increase in publication output and international collaboration over time.^28-30^ However, most studies remain observational in design, with limited high-quality or interventional research.^28,29^ Confirming our findings, authorship equity was highlighted as a concern, with many studies still led by HIC institutions and under-representation of LMIC researchers in senior authorship roles.^29-31^ Our findings highlight the level of collaboration between North-South regions, with MLW having the highest international collaboration rate, exclusively with institutions from the Global North. This was closely followed by TZN and SA, and most collaborations were North-South in nature. In addition, local authors from SA predominantly held the positions of first and last authors, whereas in MLW and TZN, these roles were typically occupied by individuals from external institutions, indicating potential publication dominance and power imbalances in North-South collaborations. As low and low-middle income countries, MLW and TZN may rely more heavily on HIC partners to publish research findings due to limited resources. However, this reliance typically decreases as countries advance economically and strengthen local research capacity. This also reflects progress towards greater independence and sustainability in research. Collaborative research between HICs and LMICs advances global health by providing funding, infrastructure, technical training, mentorship, and greater opportunities for publication, dissemination of scientific knowledge, and data support.^8,19,26,32,33^ While collaboration with HIC partners offers valuable resources and expertise, research should ultimately be locally led, addressing local priorities, ideally without reliance on external leadership, while developing local research infrastructure and support.

Collaboration and coauthorship was further highlighted by the construction and visualization of bibliometric institutional networks in each country. This enabled the identification of key collaborating institutions, which can be used to forecast future directions and target clusters to enhance South-South and North-South collaboration practices. A cross-country comparison revealed that SA maintains a well-developed institutional research network with robust domestic and international partnerships but limited regional outreach. MLW demonstrated strong North-South linkages with growing recent activity but lacked broader African engagement. The major MLW institutions were located in the Central Region of MLW, and more specifically, centred in or around Lilongwe and Blantyre, which are the two main cities associated with the country’s medical and academic infrastructure. TZN’s network was relatively underdeveloped, with few institutions involved and minimal collaboration beyond bilateral partnerships with selected USA institutions. The two major TZN institutions each covered a major region of the country (Coastal vs Northern). Visual representation has several benefits, including sharing collaboration partners and impact of output within the field of MSK trauma for future funding applications, and to support research policy, showing that researchers in the field collaborate both internally and externally.^34^ This supports findings that most SSA research output originates from SA, where MSK research is likely more well-resourced. This presents an opportunity to strengthen regional collaboration by sharing resources, expertise, and mentorship across countries. Leveraging both SA’s infrastructure and the benefits of North-South partnerships, such as capacity building and funding access, can help to develop more sustainable MSK research ecosystems in the region.

Our study has limitations. First, only two major databases were accessed for analysis, which primarily index peer-reviewed literature. Therefore, articles from non-indexed orthopaedic journals may have been missed. To address this limitation, we cross-checked with major researcher profiles within each country using ResearchGate. Another limitation was focusing on English-language articles only, which may have led to under-representation of high-quality articles published in other languages from certain regions. Furthermore, the analysis was limited to research outputs from three countries, which may restrict the generalizability of our findings to other countries within SSA. While bibliometric analyses offer a useful quantitative measure of research output, future assessments could be strengthened by incorporating additional metrics such as journal indicators, grant income information, research supervision, as well as qualitative measures including research impact, authorship roles, and open access practices.

In conclusion, this study demonstrates a steady increase in MSK trauma research output across MLW, TZN, and SA over the past decade, with growing publication volume and international collaboration remaining largely HIC-led and descriptive. As national income levels rise and countries develop, local authorship becomes more common and reliance on external collaborators decreases, highlighting that progress towards greater research equity and sustainability is achievable. Strengthening locally led MSK research remains essential to address the burden of injury in SSA. Future efforts should focus on targeted investment and regional coordination to build sustainable research capacity and inform context-specific policy and practice.

**Take home message**

- Musculoskeletal injuries are a major health burden in Sub-Saharan Africa, but research output remains limited and often led by high-income country partners.

- This study analyzed ten years of musculoskeletal injury publications from Malawi (low income), South Africa (upper-middle income), and Tanzania (lower-middle income), showing steady growth in output but continued reliance on external collaborators, especially in lower-income settings.

- Authorship was more frequently locally led in South Africa, reflecting stronger national research infrastructure and investment associated with higher economic development. These findings highlight the need to strengthen local research leadership and regional collaboration, especially in lower-income countries and progress towards greater research equity and sustainability is achievable.

- Building sustainable capacity and fostering equitable partnerships are essential to generating contextually relevant evidence that can inform policy, guide practice, and ultimately improve patient outcomes.

## Data Availability

The data that support the findings for this study are available to other researchers from the corresponding author upon reasonable request.
